# Estimating burden of syphilis among men who have sex with men

**DOI:** 10.1016/S2214-109X(21)00449-6

**Published:** 2021-12

**Authors:** R Matthew Chico, Motoyuki Tsuboi, Jayne Evans, Ella P Davies, Jane Rowley, Eline L Korenromp, Tim Clayton, David Mabey, Melanie M Taylor

**Affiliations:** Department of Disease Control, London School of Hygiene & Tropical Medicine, London, UK; Department of Disease Control, London School of Hygiene & Tropical Medicine, London, UK; Department of Disease Control, London School of Hygiene & Tropical Medicine, London, UK; Department of Disease Control, London School of Hygiene & Tropical Medicine, London, UK; London, United Kingdom; Avenir Health, Geneva, Switzerland; Faculty of Infectious and Tropical Diseases, and Department of Medical Statistics, Faculty of Epidemiology and Population Health, London School of Hygiene & Tropical Medicine, London, UK; Department of Clinical Research, London School of Hygiene & Tropical Medicine, London, UK; Centers for Disease Control and Prevention, Division of STD Prevention, Atlanta, GA, USA

## Authors’ reply

Can a change in venues sampled in syphilis serosurveys influence prevalence estimates among men who have sex with men (MSM), and has this contributed to spurious trends of decline in the past decade? In this issue of *The Lancet Global Health*, Ting-Ting Jiang and colleagues in their Correspondence pose this question and note evidence from China that HIV prevalence evaluated among MSM at public bathhouses and saunas is consistently higher than among MSM recruited through internet sites.^1^ This difference in testing venues could extend to syphilis, although such a difference was not apparent in our recent global systematic review and meta-analysis of syphilis prevalence among MSM published in *The Lancet Global Health*.^2^ In our study, the pooled prevalence estimate of syphilis among studies that recruited MSM at venues including bathhouses and saunas, clubs, and one-off public events was 6·1% (95% CI 3·7–9·1; 13 229 MSM; 29 data points). When taking into account MSM studies that used a variety of convenience sampling methods, including internet advertising, the pooled prevalence estimate was 8·7% (95% CI 7·6–9·9%; 109 065 MSM; 64 data points). Neither of these subgroup estimates were meaningfully different from our overall pooled estimate of 7·5% (95% CI 7·0–8·0; 606 232 MSM; 345 data points). However, a prevalence data compilation and trend estimation of syphilis in Yunnan province, China did find prevalence among MSM (or female sex workers) to be systematically lower in routine annual surveillance surveys and higher in research studies.^3^ The venues involved might have contributed to this difference, although neither dataset (nor their weighted sum) showed a statistically significant upward or downward prevalence trend over 2010–17; these findings do highlight the importance of inferring time trends only within series of methodologically comparable samples.

There is a need to continue harmonising prevalence-data collection and reporting of syphilis and other sexually transmitted infections (STI) among MSM. Syphilis prevalence might vary by venue and over time, the interactions of which might be best quantified and adjusted if common methods and protocols are used for screening and evaluation. These adjustments can take the form of adapting elements from the survey protocol published by WHO to test for gonorrhoea and chlamydia among pregnant women in antenatal care clinics,^4^ as Jiang and colleagues suggest. Countries and national HIV and STI programmes are encouraged to continue screening for active syphilis among MSM (and female sex workers) and reporting data through the UNAIDS Global AIDS Monitoring system. Notably, we used syphilis prevalence data from 67 Integrated Bio-Behavioural Surveillance surveys provided by UNAIDS in our meta-analysis.^2^ Efforts to harmonise data collection and reporting is as important now as ever with the aim of reducing worldwide syphilis incidence by 90% between 2018 and 2030, as proposed in the WHO’s Global Health Sector Strategy for STI control.^5^
